# Stakeholder-informed recommendations for patient-centered medication service delivery for stimulant use disorder: a qualitative study

**DOI:** 10.1186/s13722-026-00678-y

**Published:** 2026-05-27

**Authors:** Enya B. Vroom, Erin P. Finley, Jennifer Sharpe Potter

**Affiliations:** 1 Center for Research to Advance Community Health, Long School of Medicine, The University of Texas Health Science Center at San Antonio, San Antonio, TX United States of America; 2 Be Well Institute on Substance Use and Related Disorders, Long School of Medicine, The University of Texas Health Science Center at San Antonio, San Antonio, TX United States of America; 3 Department of Medicine, Division of General Internal Medicine, Long School of Medicine, The University of Texas Health Science Center at San Antonio, 7703 Floyd Curl Dr, San Antonio, TX 78229 United States of America; 4 Department of Psychiatry and Behavioral Sciences, Long School of Medicine, The University of Texas Health Science Center at San Antonio, San Antonio, TX United States of America; 5 Center for the Study of Healthcare Innovation, Implementation, and Policy (CSHIIP), Veterans Affairs Greater Los Angeles Healthcare System, Los Angeles, CA United States of America

**Keywords:** Stimulant use disorder, Medication delivery, Implementation strategies, Qualitative research

## Abstract

**Background:**

Although stimulant use disorder (StUD) is on the rise in the United States, few receive treatment. There are no FDA-approved medication treatments, despite evidence of clinical efficacy. Substance use treatment occurs within a complex landscape, with implementation of medication treatments requiring coordination among diverse partners and careful consideration of individual, organizational, and societal-level influences. The aim of this study was to identify partner- and theory-informed implementation strategies to support widespread adoption of medications for StUD.

**Methods:**

Seven focus groups and four interviews were conducted with patients and providers in diverse substance use treatment settings (12 patients, 13 providers; 25 total participants). Rapid qualitative analysis was used to distill and compare findings across patient and provider samples, and findings were critically reviewed for alignment with the Expert Recommendations for Implementing Change taxonomy in accordance with recommendations for mapping implementation strategies.

**Results:**

Participants highlighted multiple strategies that were categorized into three classes: capacity-building, integration, and implementation processes. Capacity-building strategies included training and education for providers and patients, families, and communities to reduce stigma, improve the patient-centeredness of care, and increase medication demand. Integration strategies focused mainly on organizational-level strategies such as mandating change, modifying record systems, and adding peers to care teams to enhance patient-centered care through required training, use of treatment plans, and preparing patients to be active participants in their treatment. Implementation processes strategies focused on the need for team meetings and internal/external networking to reduce siloed work among care teams and improve communication with patients.

**Conclusions:**

We report on a model for incorporating patient and provider perspectives and considering multilevel contextual factors to design implementation strategies for feasibility and acceptability across diverse SUD treatment settings. Findings highlight the importance of patient-centered approaches to medication delivery, particularly for StUD, to increase treatment engagement and adherence.

**Supplementary Information:**

The online version contains supplementary material available at 10.1186/s13722-026-00678-y.

## Introduction

In the “fourth wave” of the overdose crisis this century, marked by a shift to polysubstance use (e.g., psychostimulants and fentanyl) [1], the vast majority of overdose deaths have occurred from stimulants alone or in combination with opioids [2–4]. More than 4.3 million people were diagnosed with stimulant use disorders (StUD) in 2023 [5], and there were more than 50,000 stimulant-related deaths in 2024 [6].

Despite concerning stimulant-related morbidity and mortality, few individuals with StUD receive treatment [7]. A major barrier is the absence of FDA-approved medications [8]. StUD treatment rates are especially low compared to opioid use disorder (OUD), where medications have become a standard of care [9]. Research on effective StUD treatments also lags behind that for opioids [1], and the limited evidence-based options that exist remain underused. For example, contingency management, supported by decades of robust evidence for treating cocaine and methamphetamine use disorders, is still rarely implemented due to stigma (e.g., it can be seen as rewarding drug use), cost, and implementation challenges [4, 10]. Recent studies have shown promising evidence for the clinical efficacy of a naltrexone plus bupropion combination and other monotherapies such as mirtazapine, in treating StUD [11–14]. Additionally, the American Society of Addiction Medicine/American Academy of Addiction Psychiatry’s (ASAM/AAAP) clinical practice guideline for StUD now includes considerations for prescribing off-label pharmacotherapies when indicated [15]. Over two decades of efforts to implement medications for OUD and alcohol use disorder (AUD) in real-world practice have illustrated significant and persistent challenges to achieving routine delivery of medications for substance use disorders (SUDs) [16–19]. As research on the efficacy and effectiveness of medication for StUD progresses, it is crucial to strategize for implementation earlier along the translational science spectrum – spanning from lab science and early clinical trials to real-world practice and population health impact – to accelerate the delivery of medication for StUD [20, 21].

To inform the development of medication care models for StUD, our team conducted a qualitative implementation preparation study [22] that examined barriers and facilitators to StUD medication delivery (Phase 1) and elicited patient- and provider-informed guidance for future implementation research, including recommendations for implementation strategies to facilitate rollout efforts (Phase 2) [23]. Engaging individuals with StUD can be difficult due to rapid onset and co-occurring physical and mental health issues, making screening, clinical presentation, and treatment more complex. Additionally, social determinants of health (SDoH; e.g., homelessness, lack of insurance, unemployment) can cause variability in individual outcomes [8, 24, 25], leading to decreased access, engagement, and adherence [26–29]. There has been limited exploration of strategies to improve StUD medication service delivery in SUD treatment settings (e.g., specialty care, primary care, behavioral health). We now have sufficient evidence to justify medication for StUD as a foundational treatment approach [30], creating an urgent need to develop strategies to support timely adoption and implementation [31]. Therefore, the current paper focuses on Phase 2 study findings, which explored patient and provider perspectives on implementation supports needed to overcome existing challenges in delivering medication for StUD, with the goal of identifying partner- and theory-informed implementation strategies to accelerate the adoption of medications for StUD in future research and scale-up.

## Methods

### Study overview

The current paper extends from a two-phase study titled, “Rapid Implementation of a Novel Evidence-Based Practice to Address a Public Health Emergency,” supported by the University of Texas Health Science Center at San Antonio’s Clinical and Translational Science Award funded through the National Center for Advancing Translational Science. In Phase 1, we conducted 40 interviews with patients and providers (20 patients, 20 providers) to identify barriers and facilitators to medication adoption, which were then mapped to the Health Equity Implementation Framework (HEIF) [32, 33]. We drew on the Health Equity Implementation Framework (HEIF) due to its focus on implementation determinants across the innovation (i.e., medication), patient and provider (recipient) factors, clinical encounter, and service delivery settings and broader contexts, including health systems and society, helping us better characterize readiness and adoption challenges. HEIF provides a systematic framework for categorizing standard implementation challenges and drivers of disparities. Phase 1 interviews [23] revealed that breakdowns in patient-provider communication and therapeutic alliance often hinder the clinical encounter, highlighting the need for patient-centered StUD treatment modalities. Participants also discussed significant challenges across inner, outer, and societal contexts related to stimulant-related stigma, treatment costs, and concerns related to medication side effects or lack of FDA approval, underscoring the need for training, education, and cost-navigating strategies. Overall, participants were generally supportive of medication for StUD treatment, especially given the prevalence of stimulant use and StUD they are experiencing in their communities. Details and findings from Phase 1 are reported elsewhere [23].

Phase 2 is the focus of the current paper and reports on patient- and provider-generated strategy recommendations. After Phase 1 findings were mapped to the HEIF, in alignment with recommendations from the Expert Recommendations for Implementing Change taxonomy [34] for mapping implementation strategies to specific constructs within implementation frameworks, we compiled a list of strategies to address identified barriers. These mappings informed the development of the semi-structured interview guide used in Phase 2 to solicit feedback on proposed implementation strategies.

### Participants

Participants included patients with a history of SUD and providers currently treating patients with SUD, including prescribing providers and other care team members (e.g., clinical directors). The study employed both longitudinal purposive and snowball sampling techniques for recruitment. First, we invited participants from Phase 1 to participate in follow-up focus groups via email or phone. For providers, Phase 1 recruitment involved distributing study information through the authors’ institutional listservs, including an established network of substance use treatment organizations in Texas and attendees of a national harm reduction, hospital opioid use disorder treatment, and medications for SUDs Extension for Community Healthcare Outcomes (ECHO) series (> 500 registrants nationwide). Providers included physicians, nurse practitioners, clinical directors, and counselors working in organizations delivering SUD treatment in outpatient and inpatient settings, including primary care, addiction specialty care, and integrated care. To capture a broad range of perspectives relevant to strategy recommendations, we intentionally sampled multidisciplinary providers and other care team members, including prescribing and non-prescribing providers.

Patient recruitment included distributing study information through providers’ organizations and the same institutional listservs used for provider recruitment, where providers were asked to share study information with their patients. Existing patient and provider participants also referred others to the study. To account for polysubstance use, to overcome barriers to recruitment within a historically hard-to-reach population (i.e., highly stigmatized), and to capture broad perspectives among patients with experience or direct exposure to stimulants, we did not require a formal StUD diagnosis for inclusion. Patient participants were eligible for participation if they had a current or prior SUD.

In total, we had 17 returning participants and eight new participants from Texas, Pennsylvania, Louisiana, and Illinois, with most participants residing in Texas (88%) for Phase 2 data collection. This study was reviewed and approved by the UT Health San Antonio’s Institutional Review Board. All participants received a $50 gift card for their participation.

### Data collection

**Fig. 1:**
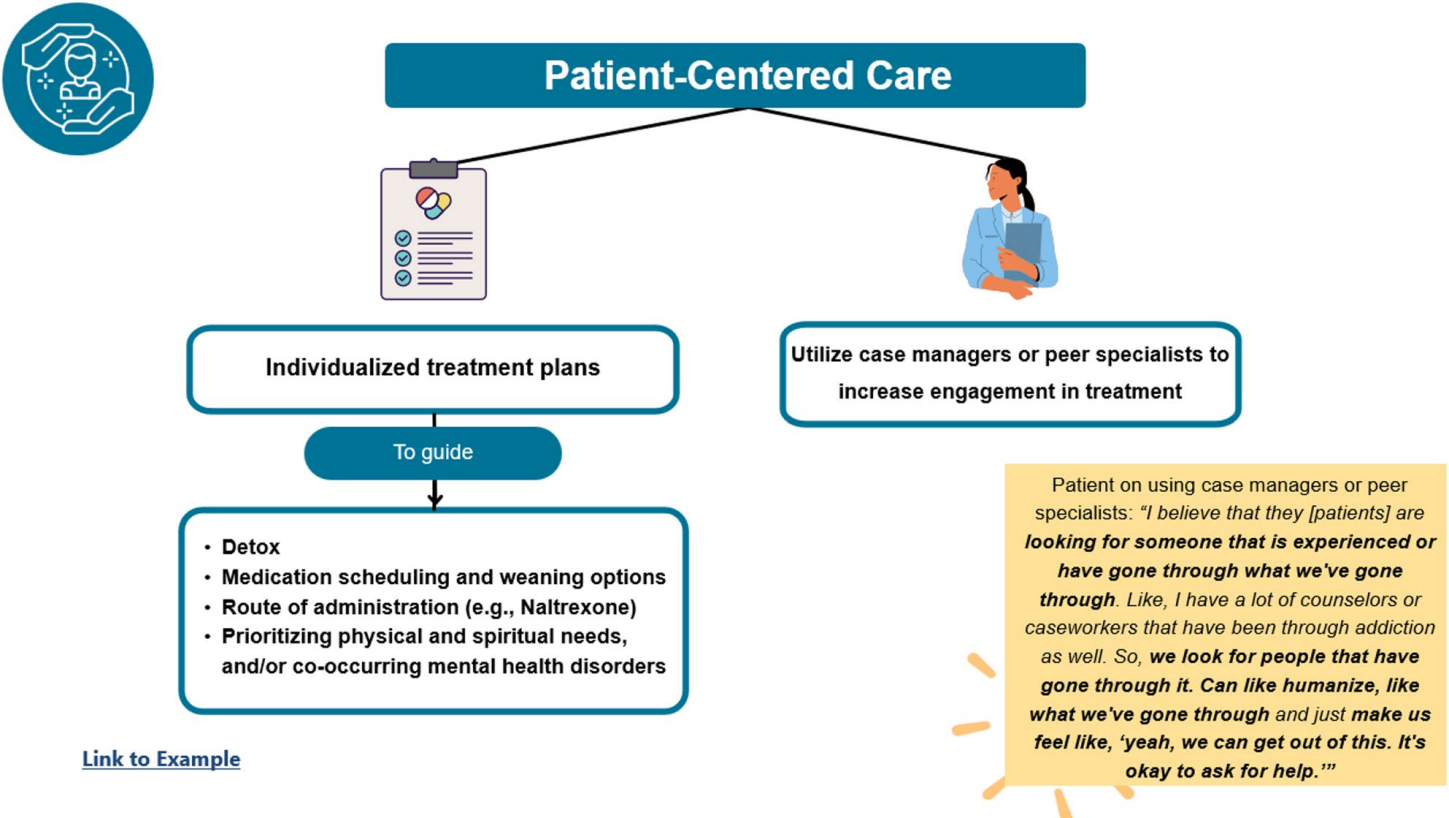
Example visuals accompanying the qualitative guide

To elicit participant perspectives, we conducted 7 focus groups (4 with patients, 3 with providers), with an average of 3 people per group (range 2–6). In a few instances, only one individual attended the group; in these cases, we conducted in-depth individual interviews (3 with patients, 1 with a provider) using the same semi-structured guide to maintain consistency. In total, 25 individuals (12 patients, 13 providers) participated. To determine the study’s sample size, we drew on estimates from research suggesting data saturation can be reached within two to six focus groups (per subgroup, e.g., patients or providers) using a semi-structured guide among a relatively homogeneous population [35, 36]. Focus groups and interviews were conducted between November 2024 and April 2025, recorded with participant consent, and held virtually on Zoom, a HIPAA-compliant platform. On average, focus groups lasted approximately 60 min and interviews lasted approximately 45 min and were conducted by the co-principal investigator (Co-PI) and two trained qualitative research staff. The semi-structured guide included open-ended questions to elicit participants’ perspectives on recommendations and feedback on strategies for overcoming barriers, including education and training (e.g., topics, focus areas, ideal methods of delivery, target groups), approaches to patient-centered care (e.g., individualized treatment plans, clinical encounter), and cost/funding (e.g., sources). The guide was also paired with a PowerPoint presentation that featured visual examples of potential tools for patient or provider education (e.g., informational websites) and individualized treatment plans (e.g., the U.S. Department of Veterans Affairs [VA] Personal Health Inventory) [37] to gain targeted feedback for implementation strategy selection and tailoring (Fig. 1). Patient and provider guides covered the same overarching topics to facilitate comparisons across groups, with slight variations in question wording to ensure clarity and accessibility [38]. PowerPoint visuals remained the same across groups. Please see Additional file 1 to view the semi-structured guide.

### Data analysis

Guided by the Planning for and Assessing Rigor in Rapid Qualitative Analysis (PARRQA) framework [39], we used a rapid qualitative approach, supported by structured summaries of transcribed data, to analyze focus group and interview data. This validated approach aligns with the goals of the current study by efficiently converting findings into actionable insights to guide future implementation research [40–42]. Shortly after each data collection session, transcriptions were reviewed for accuracy, and trained analysts developed structured summaries for each transcript. The summary template was organized by topic domains covered in the focus group/interview guide (e.g., education/training, approaches to patient-centered care, and cost/funding) and allowed for the distillation of emergent findings. The template also included a section dedicated to memoing, which we used as a consistent prompt for reflexivity discussions, including emerging interpretations, reflections on assumptions, and power dynamics. The study’s Co-PI reviewed all summaries, and the research team met weekly during data collection and analysis to ensure consistency and completeness in approach and to discuss and resolve any disagreements collaboratively. To facilitate a comprehensive review of the findings, the analysts compiled summary content into two matrices (one each for patients and providers), also organized by topic domain. Findings were consistent across focus groups and interviews and were therefore analyzed together. Between January 2025 and June 2025, the analysts summarized data related to each topic domain into narrative summaries. These were discussed among the entire research team and refined, which included constant reference back to the matrix, summaries, and, when necessary, the original transcripts for verification and to enhance the validity of synthesized findings [40]. When narrative summaries were complete, the team reviewed and compared findings within and across patient and provider samples, critically reviewed participant recommendations for alignment with ERIC implementation strategies, discussed discrepancies, and built consensus on key strategies, in accordance with recommendations for developing a multicomponent implementation approach [43, 44]. The resulting list of ERIC strategies was refined and sorted into classes (i.e., strategies related to capacity-building, integration, and implementation processes) based on recommendations from Leeman and colleagues [45] to increase pragmatism and appropriateness for community-based settings.

## Results

Below, we discuss patient- and provider-recommended strategies for adopting and implementing medications for StUD treatment, organized by their emphasis on capacity-building, integration, or implementation processes (i.e., classes; see Table 1). For clarity, strategies are named according to the ERIC taxonomy in bolded headers and italicized text. Additionally, several strategies naturally intersect or complement each other across classes. Table 1 outlines cross-cutting strategies across classes.

**Table 1:** Theory- and stakeholder-informed selected implementation strategies for StUD medication adoption and implementation

ERIC Strategies by Classes	Cross-cutting ERIC Strategies & Corresponding Classes	
**Capacity-building Strategies**	**Integration Strategies**	**Implementation Processes Strategies**
• Conduct educational meetings for providers	• Facilitate relay of clinical data to providers	--
• Ongoing training for providers	--	--
• Develop and distribute educational materials for providers, patients, families, and communities	• Change record systems by adding quick access resources to EHR systems	--
• Learning collaboratives	--	--
• Capture and share local knowledge	• Use of mass media to share patient and provider testimonials	--
• Obtaining and using feedback from patients and providers	--	--
• Academic partnerships	--	--
**Integration Strategies**	**Capacity-building Strategies**	**Implementation Processes Strategies**
• Mandate change	--	• Involving executive boards
• Creating or changing credentialing/ licensure standards • Organizational credentialing requirements (internal) • Professional certification/licensing requirements (national/governmental)	--	--
• Create new clinical teams • Peer recovery support specialists • Case managers • Mental health clinicians • Preparing patients to be active participants in their treatment	--	• Involving patients/families in their care
• Change record systems • Individualized treatment plans • Develop and implement tools for quality monitoring	--	--
• Change service sites • Telehealth • Medication delivery service	--	--
• Use mass media • Increase patient demand	• Developing and distributing educational materials • Social media	--
• Financial strategies • Access new funding • Fund and contract for the innovation • Make billing easier	• Obtaining and using feedback from patients, providers, and the community	--
**Implementation Processes Strategies**	**Capacity-building Strategies**	**Implementation Processes Strategies**
• Organize team meetings	--	--
• Promote network weaving	--	--
• Obtain commitments • Formal & informal	--	--

### Capacity-building strategies

#### Conduct educational meetings and ongoing training for providers

Both patients and providers noted that training providers, including addiction medicine and general practitioners (e.g., primary care, family medicine), is critical to overcoming barriers in the clinical encounter and to bolstering clinician knowledge and confidence in adopting medications for StUD treatment. Providers discussed that training should be asynchronous, self-paced, and, if possible, provide continuing education credits. Both providers and patients emphasized that training should be comprehensive, including topics such as stigma, medications for StUD and how to discuss medication options with patients, current evidence and pharmacokinetics/dynamics of StUD medications (i.e., *facilitate relay of clinical data to providers*), patient social determinants of health (SDoH), baseline insurance-related information (e.g., billing practices), and patient-centered approaches, including individualized treatment, shared decision-making, and therapeutic alliance. One provider noted, “*I think [training] should be robust. It needs to be*,* I think*,* more than a YouTube video. Not that there’s no value in that*,* but I think robust training is really important.”* Provider and patients both emphasized the importance of ongoing training for providers, noting that “a few hours of training” is insufficient, and that providers may struggle to engage in training on their own.

#### Develop and distribute educational materials for providers, patients, families, and communities

We asked participants to provide feedback on best practices for developing and distributing educational materials via a website, based on Phase 1 interview findings that highlighted the need for quick access to resources. Providers thought that a provider-focused website highlighting StUD training opportunities, patient education resources, quick-start guides, medication guidelines, funding opportunities, and access to communities of practice (*learning collaboratives*) would be a helpful resource, especially for non-addiction specialists. Providers had mixed views on whether a website would be a good educational tool for patients. Some provider groups noted that simple visuals outlining treatment options and resources would be valuable, while others were concerned about patient technology literacy and limited internet access in rural areas.

Patient groups were generally supportive of an educational website, emphasizing that the website layout should be clear, concise, and easy to navigate to avoid overwhelming patients and families. Patients discussed the usefulness of including practical tools or links for finding treatment options (e.g., zip code finder), cost navigation, information about StUD medication, side effects, SUD medication myths, and recovery resources (e.g., local recovery meetings, hotlines). Additionally, patients noted that if a website is going to be used to educate families and/or communities with the goal of reducing stigma, it is ideal to focus on direct, fact-based information about StUD, available treatment options, including medication, and how they can “support a loved one suffering from addiction.” One patient group discussed that direct, strength-based messaging, rather than “emotional appeals,” would be a more effective strategy to reach family and community audiences.

For family and community… less focusing on the war stories of, ‘I was out there smoking crack, or doing dope,’ [more of] ‘Look, I have been able to overcome this and live as a productive member of society, and this is my story.’ More focused on the strength and hope. ‘I’m a productive member of society. I have a full-time job. Got my kids back.’ Showing more of that versus people using. – Patient Focus Group #2.

Another patient suggested the website should include talking points to assist patient discussions with family about medication treatment, stating, “*…when their family starts questioning ‘em*,* they know what to say. They don’t just say*,* ‘I’m just taking this because it helps*,*’ but they actually know the correct way to answer the questions.*” Additional educational materials discussed by patient and provider groups included pamphlets placed in patient waiting rooms and quick guides for providers located in electronic health record (EHR) systems (*change record systems*) that review treatment guidelines or have information that can be printed for patients.

#### Capture and share local knowledge

Both groups emphasized the importance of including testimonials (quotes or videos) from patients and providers that highlight the successes of StUD medication adoption and implementation in all educational materials, as this is essential for building patient trust and provider buy-in through the *use of mass media* (see also Integration Strategies).

…cause I know I would wanna hear, like I said, some providers haven’t gone through the addiction. They’re not on maintenance. They’re just giving you basic knowledge. If, as a patient to a patient and they can give testimonies… I think it would be a better understanding patient to patient so that they can get a general idea of everything. – Patient Interview #3.

#### Building academic partnerships

We asked providers how researchers could support treatment organizations and providers in communicating with patients about StUD treatment options and cost. Providers discussed the role academic partnerships could play, including by facilitating training and creating an up-to-date, centralized database of funding sources for StUD. Providers also suggested that academic researchers could assist by offering strategies to improve patient treatment access, conducting communication campaigns to address societal stigma, and examining and disseminating findings to support continued advocacy for medications for StUD.

…providing something that is in writing with the findings, like how important it is to stay on injectables or something like that. That way [providers/organizations] have something to bring when they are advocating. Let’s say they want to advocate at the state level or whatever… how you have this PowerPoint, where it has everything right there, I think that could be really helpful. I also think if you had a page that they could find those resources, like, “Hey, these companies offer treatment for free. These companies offer it for this type of cost.” If it was something that was more accessible for them rather than them having to scroll all over Google. – Provider Interview #1.

### Integration strategies

#### Mandate change

In addition to supporting continuing education credits, providers suggested that organizations should mandate StUD-related training for internal credentialing and should consider incorporating training into existing meetings or protected time to enhance its feasibility and perceived value. Providers reported that “top-down” buy-in to support the implementation and sustainment of training programs (*involving executive boards [decision-makers])*, including both initial and ongoing training, is essential for standardizing and mandating training.

If an organization and its leadership are invested in this, then rolling this kind of training out is pretty straightforward because it’s just part of the organization’s philosophy or what they’re gonna offer versus places where they’re still maybe not on board, they’re not gonna roll out any kind of training, and so you really have that top-down issue… – Provider Focus Group #2.

Creating or changing organizational credentialing standards to require onboarding providers to complete StUD-specific training may be particularly important for primary care and emergency medicine providers, as participants reported that they are often the first point of contact for intervention and treatment initiation. More broadly, providers discussed a notable gap in healthcare professional education and training related to addiction and patient SDoH, which they described as fueling stigma within the U.S. healthcare system and leaving providers underprepared to address SUDs, especially StUD, in practice. One provider noted, “*It’s a little bit frustrating to have to see people affected by the stimulant use disorder and not having as many tools that we would like to be able to treat them.*” Some providers highlighted the need to alter initial and continuing education at a national level.

#### Create new clinical teams

Both patients and providers discussed the value of embedding peers with lived experience within care teams to assist with patient engagement, education, and social/healthcare service systems navigation. Both participant groups stressed that a holistic approach to care can be more effectively facilitated by peer recovery support specialists and/or case management through better coordination of care, addressing certain SDoH (e.g., housing, clothing, employment), and creating/monitoring treatment and/or recovery plans, thereby improving treatment engagement and adherence and reducing burden on physicians and/or therapists. One provider stated, “*We have peers in a couple of our treatment programs for adults*,* and then we also have peers in our youth programs*,* and so that’s an avenue*,* either to get people started into treatment and/or during treatment*,* to keep them – help keep them engaged…*” Providers and patients discussed that peers can also serve as a bridge to bolster social support by *educating and involving families*, advocating for necessary services/resources, and better *preparing patients to be active participants in their treatment* through education on StUD treatment options. Additionally, a provider mentioned the benefit of adding a licensed mental health professional to their team to assist with addressing co-occurring mental health challenges, a common barrier to treatment engagement.

#### Change record system

Phase 1 interview findings revealed the need for individualized care and use of tailored treatment plans to overcome barriers in the clinical encounter. Participants were asked to review an example individualized treatment plan, the VA’s Personal Health Inventory [38], and share whether they thought utilizing such a tool would be helpful in treating StUD and what would be needed to make it more compatible. Generally, patients agreed the tool would be useful, as it would consider goals not only for StUD but also for physical and mental health care, addressing the “*full scope of a person’s life and recovery needs.*” Patients appreciated a treatment plan that requires personal reflection and goal setting, breaks treatment into manageable steps, and has the potential to provide structure during the transition from inpatient to outpatient treatment, noting this would assist with engagement and understanding treatment expectations.

Providers were also supportive and noted the potential of an individualized treatment plan to facilitate patient-provider conversations, track progress, and enhance patient motivation.

I think the easiest way, if we were going to try to get information from provider to patient would be through the electronic health record, and so we have patient education notes that we can add on various topics, and just as we were closing out the note, it’s super fast, and we can customize it to the patient’s needs. – Provider Focus Group #1.

Two providers discussed using similar tools in their practice, which have been customized to fit their workflows (i.e., exist within their EHR system). Another provider discussed how they use treatment plans to track patient outcomes and progress, enabling them to adjust protocols when patient needs are not being met. Their clinic also uses the data to showcase successes and improvements for existing or potential funders. Both patients and providers reported that treatment plans can assist with tracking progress and goals over time, which participants described as essential for adapting plans in response to evolving needs (i.e., *develop and implement tools for quality monitoring*).

We started a brand-new clinic. I have never had experience running a clinic before. We’ve realized, ‘Okay, well, how are we monitoring this?’ Especially with donors. Donors want to know, ‘What are your outcomes looking like, so we know if we’re gonna keep funding this.’ Also, we wanted to look at, ‘are we doing everything to keep our participants in treatment, in [medication] treatment, specifically?’ We did start to lose a couple people, and we wanted to know, ‘is it us?’ That’s why we started implementing [treatment plans]. – Provider Interview #1.

Patients recommended that treatment plans be completed with their providers, while providers suggested that plans could be self-administered or completed collaboratively in session. To improve compatibility, both groups emphasized that treatment plan templates and prompts should be simple, concise, and straightforward to account for low literacy levels. Patients also recommended adding a religious or spiritual component to treatment plans, noting its relevance for certain faith-based recovery pathways, and incorporating information on stimulant use/StUD to facilitate shared decision-making and patient education.

#### Change service sites

Both patients and providers discussed the limited availability of SUD treatment and recovery services in rural areas, as well as patients feeling emotional or physically overwhelmed by treatment logistics or the treatment setting, which can hinder access and engagement.

I think that the previous experiences or just what might be triggered for a patient who goes to the clinic and feels very anxious all of a sudden because of that experience, and not associating that with getting better, necessarily. I’ve had patients who’ve come, and then they had to leave ‘cause it was just too much. – Provider Focus Group #3.

To increase access, telehealth options can provide more flexibility for appointments and transportation, as well as direct medication shipping to the patient (when applicable), which can also reduce barriers to medication retrieval.

Our clinic does a lot of telemedicine… we have agreements and contracts with [insurer] for mail out prescriptions, and so there are patients that don’t even need to leave their house… we’ll send a DocuSign to email or their text messages, and they can sign it right there… They can do everything via video, so they don’t have to move or leave. It’s just a very seamless process. I think makin’ it as easy as you can for them… it helps them get increased access. – Provider Focus Group #1.

#### Use mass media

To address stigma and increase awareness of treatment options, both patients and providers reported that using social media (e.g., TikTok, Facebook) or commercial marketing techniques to disseminate educational content about StUD and treatment options can help engage patients, families, and community members. One patient noted, “*Because a lotta people*,* when they’re using*,* their either on their phone*,* on the computer*,* watching TV*,* mindlessly scrolling. The offer for assistance pops up in the middle of it*,* could actually catch ‘em when they need it the most.”*

You know how Facebook has those random ads that pop up? Are you suffering from this or whatever? I know it sounds cliche or weird, but I feel like those people probably aren’t going to doctor’s appointments. They’re probably not going to school. They’re probably not going to those places. At that point, once they’ve already crossed over into addiction, it will have to be introduced in a way that is like a commercial or an ad or something like that. – Patient Focus Group #3.

One provider group also discussed using social media influencers with large followings to spread educational information. Patients discussed that *developing and distributing educational materials* and using mass media such as commercial or social media advertisements can also *increase demand* by educating patients, families, and communities about potential StUD medication treatment options, and encouraging patients to discuss these options with their provider.

#### Developing financial strategies

Patients and providers were asked how they navigate SUD treatment cost, including what patients find helpful and how providers and treatment organizations can assist patients with StUD medication costs. Providers believe that care teams and treatment organizations have a responsibility to advocate for StUD-specific funding, especially at the state level. They noted advocating for funding opportunities that align with community needs is particularly important for StUD treatment, given the precedence that has been established for OUD treatment (*access new funding* & *fund/contract for the innovation*).

I think when it comes to StUD, which is starting to become a big problem, which we don’t have really good options for still, but we also don’t have any big funding sources to tap into at the moment. You have to actually look for small little crumbs here and there, but unlike OUD, where there’s a lot of money floating and it’s easy to access money, they’re trying to find people who can treat OUD the right way. Everyone’s ready to give money. They’re just trying to find the right people. The meth use disorder, it’s a little different story. You have to search for places where you can tap into to get some money for these treatment options. – Provider Focus Group #2.

Providers also felt they had an obligation to be better informed about how services are paid, how to advocate for patients within payer systems, and how to provide guidance to patients regarding insurance and costs. Similarly, patients thought providers should be more proactive in sharing financial resources and/or planning for the potential loss of insurance coverage.

I personally just received a phone number of one or two people that can assist with the Marketplace…. Getting that information to clients would be huge so that they know, ‘Hey, if you call this person, you’re not gonna pay for it, but they’re gonna make sure that you’re gonna get a plan that’s gonna help pay for your treatment.’ – Patient Interview #2.

Providers discussed the challenges of navigating complex funding systems and the mandatory, often time-intensive documentation required to safeguard program funding. Additionally, funding mechanisms intended to support care for uninsured individuals frequently impose narrow eligibility parameters, creating challenges to low-barrier, patient-centered care. To *make billing easier*, providers recommended reduced documentation requirements, and, when possible, both patients and providers suggested having dedicated benefit coordinators on staff to assist with navigating preauthorization requirements, service eligibility, Medicaid enrollment, and connecting patients to insurance liaisons. Patients also recommended that treatment organizations clearly display and/or communicate accepted insurances both in office and online.

The way that [medication for AUD] is set up is much easier because it allows us to use our existing data management – our existing EHR… Allowing organizations and providers to use what they already have as opposed to requiring data entry into a separate system helps with implementation… – Provider Focus Group 3.

Yeah, ‘cause the billing specialists are the ones that really – they have to step in whenever we start going through problems… Billing specialists are definitely important to actually have at the table ‘cause they’re the ones who deal with the private insurance and with the Medicaid, with the Medicares, the billings, all of that. – Provider Focus Group #3.

A general message across both patient and provider groups was the importance of involving patient and family members in care and ensuring provider, patient, family, and community needs are considered in organizational decision-making and identification of funding opportunities (*obtaining and using feedback from patients and providers).*

I think you [asked] who should be at the table when plans are made for funding and programs?… just as many perspectives as possible across the spectrum – people that are impacted by these situations, by these conditions. That would include leadership and patients and people that run treatment programs, and people in incarcerated settings. I think statisticians, public health professionals, government officials – I think everyone’s perspective, because this is something that touches so many parts, so many people in so many different spaces. – Provider Focus Group #2.

### Implementation processes strategies

#### Organize team meetings and promote network weaving

Patients were asked to discuss their experiences with patient-centered SUD care. Patients’ experiences varied, with some noting they had never received individualized care and others stating they had only experienced it within inpatient settings, with one patient stating, “*Only at that short period of time at a rehab*,* and that’s only at some rehabs. …the ones that… say*,* for example*,* that are at the hospitals… or the ones that are free*,* there’s no way you get any.*” Multiple patients felt they had to “DIY” their recovery due to a lack of guidance.

The lack of knowledge or training [of providers] you face, talking to [primary care providers] or even sometimes [SUD] specialists about addiction recovery, it can be staggering… I feel like that’s why a lot of people fall through the cracks, because it’s so difficult to navigate these symptoms and like tailor build your own recovery path that is gonna work for you. If you’re not able to do that because of mental health issues or housing issues… people become discouraged. I just really had to learn how to speak up for myself. – Patient Focus Group #1.

Patients reported that addressing co-occurring mental health issues was key to effectively managing their SUDs, but also noted that mental health is often undertreated, especially in “free or hospital-based programs.” Providers were also asked how they incorporate patient-centered care into their practice, offering varied responses. Providers acknowledged that patient-centered care requires time and coordination across internal and external care teams, depending on the type of provider and treatment setting. They emphasized that communication among a care team (e.g., physician, therapist, or peer specialist) is essential to avoid siloed work, which can prohibit patient-centered care, including engagement. Care teams need to be aligned, as conflicting information across care team members can hinder patient-provider rapport, requiring protected time for organized team meetings and network weaving to facilitate mission alignment and collaborative problem-solving within the organization.

What ends up happening is if the patient perceives that they are getting… conflicting information, they first of all feel confused. Second, they feel stigmatized – they interpret it in all different sorts of ways. It’s very important that the message to the patient is unified. That can only happen when there are open lines of communication between all the different people that are taking care of the patient. – Provider Focus Group #2.

Additionally, some providers described incorporating patient-centered care by ‘meeting patients where they are,’ acknowledging underlying health or psychosocial factors, and tailoring the treatment approach based on what patients are ready and willing to engage in.

#### Obtain commitments

Both providers and patients discussed that forming formal or informal partnerships with entities that provide needed services and/or resources can help facilitate patient-centered, holistic care, including recovery housing, food banks, primary care, pharmacies, recovery community organizations for peer support referral, or local transportation entities. Peers were suggested as particularly well-suited to conduct outreach to facilitate partnerships with more informal entities, such as employment opportunities and clothing closets. Providers also discussed transportation solutions they have previously used, such as Lyft business accounts, to enhance patient access to appointments, lab testing, and medication retrieval.

## Discussion

To inform the development of medication care models for StUD, this study identifies implementation strategies that may accelerate the adoption of patient-centered medication delivery for StUD. It draws on both theory (HEIF and ERIC) and input from key stakeholders, highlighting recommendations that account for the multilevel context of the StUD treatment landscape. To our knowledge, this is among the first studies to generate implementation strategies for patient-centered medication delivery for StUD. Participants identified multiple strategies to be integrated as part of a comprehensive effort to broadly implement medication for StUD, including provider training, patient, family, and community education, and organizational-level process/procedural considerations. We also saw that many strategies were interwoven across classes, as one strategy may enable another. For example, mandating some form of organizational change (an integration strategy) will likely involve decision-makers such as executive boards/leadership (an implementation processes strategy).

In response to inner context barriers that emerged in prior findings (e.g., stigma, lack of training, provider hesitation to prescribing), Phase 2 findings indicate the need for dedicated implementation support for organizational leadership, providers, and staff, particularly through encouraging “top-down” buy-in to create space for initiatives such as provider training and organizational change. This aligns with prior research underscoring the influence of leadership and organizational-level factors on the acceptance and implementation of change initiatives [46–48]. To enable providers and organizations to establish sustainable models for StUD medication delivery, participants noted that external support may be required. In the outer and/or societal contexts, researchers, funders, or national organizations (e.g., federal agencies, professional associations) can contribute through interactive capacity-building strategies, including external facilitation, technical assistance, or ongoing consultation to support addressing site-specific challenges and facilitating change.

Facilitation may combine diverse strategies that fulfill complementary functions, for example, developing tools for quality monitoring (implementation processes), changing record systems to better track outcomes (integration), conducting audit and feedback cycles with providers to reinforce and monitor outcomes related to patient-centered care (capacity-building), and/or engaging organizational decision-makers in the implementation effort (implementation processes). Evidence from other research illustrates both the promise and limitations of facilitation. A recent study found that facilitation increased provision of medication for tobacco use disorder during active facilitation and post-facilitation, led to delayed increases in medication provision for AUD post-facilitation, and did not improve provision of medication for OUD in HIV clinics [49]. Differences across SUDs may have resulted from higher baseline readiness, varying levels of academic detailing (e.g., direct educational outreach), the presence/absence of program champions, or lower perceived need at baseline due to existing infrastructure [49]. These findings suggest that a bundle of strategies, provided at varying levels of intensity and intervals, may be necessary to achieve successful StUD medication adoption and that organizational capacity is a crucial consideration in planning. Needs may differ substantially for organizations starting up (no existing capacity) versus scaling (building on existing capacity). For example, organizations already delivering medications for SUDs (e.g., medication for OUD) may not require, or benefit from, the same level of engagement in strategies to accelerate adoption as those with no existing infrastructure [50]. Within the inner context, tailoring strategies to organizational capacity may be essential for developing approaches that accelerate the adoption of StUD medications and improve their sustainability in practice.

Additional strategies discussed by participants included the potential need for commitments from external entities to support holistic care (e.g., pharmacy, recovery support services), promoting network weaving, and leveraging mass media to reduce stigma at the community level. Such strategies may help address disparities in access to care and increase patient demand, critical barriers discussed within patient factors and inner and outer contexts. Partnering with community-based organizations or adopting multidisciplinary team approaches may further facilitate referrals to needed services and social supports [51], although prior research highlights that warm handoffs and follow-ups may be critical for referral success [51, 52]. Related to provider factors, expanding clinical teams to include peer recovery support services, or partnering with recovery community organizations/centers that can provide such services, also emerged as a recommended strategy. Certified peer support specialists, individuals with lived experience of SUD and recovery who are trained to provide nonclinical support, can strengthen referral pathways and connect patients to informal resources such as transportation, clothing, and housing [53], and addressing these and other SDoH-related disparities can positively impact treatment engagement and adherence [54, 55]. In addition, peers can play a key role in patient and family education about stimulant use and available treatment options, including medication.

Related to teaming and network weaving, prior research suggests that whole-team cohesion may not be necessary across all clinical team members; targeted communication may only be needed among those most directly involved in medication delivery, such as physicians and pharmacists [46]. Our findings suggest that internal team cohesion, supported by dedicated team meetings to align treatment plans, may be essential to avoid siloing and miscommunication with patients (i.e., a direct barrier to the patient-provider clinical encounter), which participants reported can undermine engagement. Further research is needed to examine how team cohesion should be structured with external partners (e.g., pharmacy), as both internal and external alignment may be important in settings where SUD medications are delivered less frequently, such as primary care.

In addition to capacity-building strategies such as multilevel education and capturing and sharing local knowledge, which have been shown to improve provider attitudes and behaviors toward medication delivery [56–58], participants emphasized the need for community-level education to dispel myths about addiction, medication, and stimulant use. Recent stigma-reduction campaigns show promise, demonstrating that communication efforts, including websites, using educational, testimonial, or advocacy-based content can reduce stigma related to OUD in societal contexts [59, 60]. However, such campaigns are most impactful when co-produced with the local community and individuals with lived experience, helping to ensure both relevance and credibility [61]. For educational websites, participants emphasized the importance of clear, strength-based messaging, with quick-access resources such as treatment options, funding sources, cost navigation, and training opportunities to overcome knowledge and access barriers among providers and patients. Our study participants also highlighted the potential of social media as a tool for disseminating educational content for patients, suggesting the use of social media advertisements and influencers (i.e., individuals with large followings on social media platforms with established reputations as trusted messengers of information) [62] to reach broader audiences. Online communities are increasingly becoming spaces where people seek health information from trusted peers, and recent research shows promising results indicating that exposure to social media influencers was associated with greater awareness and more favorable views of harm reduction (e.g., syringe exchange) among the general public in West Virginia [63]. However, the use of mass media approaches, such as social media, should be approached cautiously, given concerns about misleading or predatory marketing practices in the SUD treatment landscape [64]. Mass media efforts should prioritize accurate, evidence-based information and support patients in identifying high-quality, ethical care.

In addition to theory- and best practice-guided strategy identification, incorporating end-user perspectives on barriers, facilitators, and strategy recommendations (e.g., from patients and providers) is critical for co-designing future implementation studies. Prior research on strategies to expand medication delivery for AUD or OUD has shown modest results, which may be due in part to limited integration of provider- and patient-level perspectives [65], or insufficient tailoring of strategies to better fit organizational infrastructure, target populations, or regional context [66]. In this study, the ERIC taxonomy provided a foundation for targeted, theory-informed strategy selection. Yet, recognizing concerns about the length and complexity of existing strategy lists [45], we expanded beyond the taxonomy to organize strategies into functional categories that may better reflect real-world healthcare practice. These strategies should not be viewed as exhaustive or final, but rather as a starting point for identifying, tailoring, and testing strategies to advance patient-centered medication delivery for StUD in future implementation research. Qualitative methods are valuable for providing an in-depth understanding of context to guide strategy identification. Additional research is needed to test a multilevel component implementation approach to assess effectiveness across diverse SUD service settings (e.g., primary care vs. specialty care). Mixed methods designs are recommended to balance depth of insight with generalizability and reproducibility to further accelerate translation [22].

### Limitations

This study had several limitations. Due to scheduled focus groups having no-shows, some data were collected through individual interviews rather than group discussions. Consistency in content and approach was preserved by using the same semi-structured guide and accompanying visuals across both formats. Second, we recognize that inviting Phase 1 interview participants to also participate in focus groups may limit the incorporation of new perspectives. However, this continuity was intentional to support the iterative co-development of recommendations for future implementation, built authentically on participants’ prior descriptions of likely barriers and facilitators [67]. Additionally, eight new participants were recruited, which may have helped prevent potential narrowing of perspectives. Third, most participants resided in Texas, and although many participants shared experiences from their time living or working in other states, findings may primarily reflect Texas-specific healthcare and policy contexts. Future research should explore needs, resources, and strategy recommendations for expanding implementation of medications for StUD in diverse geographic settings with differing populations, workforces, funding, and treatment policies. Fourth, we confirmed that patient participants had a current or prior SUD diagnosis, but did not collect information on the specific type of diagnosis (e.g., StUD or OUD), which may affect the interpretation and generalizability of the findings. However, recruitment of patients occurred primarily through provider referral in SUD treatment settings, and recruitment materials explicitly emphasized the study’s focus on stimulant use and StUD. Finally, the study did not directly investigate the temporality of the recommended strategies, i.e., whether some strategies would need to occur before or in tandem with others (e.g., acquiring funding before training). Future research would benefit from a deeper exploration of strategy temporality to further inform strategy sequencing and intensity.

## Conclusions

The goal of this implementation preparation study was to inform future implementation research efforts aiming to accelerate the adoption and implementation of medication for StUD treatment. Findings highlight the importance of patient-centered approaches to StUD medication delivery to increase treatment engagement and adherence. By incorporating the perspectives of patients and providers and considering multilevel contextual factors, future implementation efforts can benefit from comprehensive implementation approaches that incorporate multilevel strategies and are feasible and acceptable across diverse SUD treatment settings.

## Electronic Supplementary Material

Below is the link to the electronic supplementary material.

Supplementary Material 1

## Data Availability

The datasets generated and/or analyzed during the current study are not publicly available due to confidentiality concerns, but are available from the corresponding author on reasonable request.

## Abbreviations

StUD: Stimulant use disorder
SUD: Substance use disorder
HEIF: Health Equity Implementation Framework
ERIC: Expert Recommendations for Implementing Change
AUD: Alcohol use disorder
OUD: Opioid use disorder
SDoH: Social determinants of health
