# Supplementary material for: Stakeholder-informed recommendations for patient-centered medication service delivery for stimulant use disorder: a qualitative study
Source: Addict Sci Clin Pract. 2026 May 27;21:50. doi: 10.1186/s13722-026-00678-y (PMC13244936; doi:10.1186/s13722-026-00678-y)
Supplement: Supplementary file 1 — Supplementary Material 1 [file 13722_2026_678_MOESM1_ESM.docx]

**Phase 2 Focus Group/Interview Guide**

**Section A: Member Checking**

*The interview starts with a review of Phase 1 interview findings, presented via a PowerPoint slide deck. Start screen sharing the PowerPoint and bring up the first slide that shows the overview of the Phase 1 findings.*

1. What are your thoughts on these findings? (*Note: Make sure to take a pause here before moving on to the probe.)*

- 1. Do these ring true to you as the most important barriers to stimulant use disorder (StUD) treatment - Why or why not?
  2. Is there anything important you think is missing?

**Section B: Next Steps – Recommendations for Strategies**

*Use PowerPoint slides to present topic areas, examples, and definitions of terms, such as patient-centered care (see Figure 1).*

**Education for Providers, Patients, Family, and Community**

2.1 Here are some of the topics that we have already discussed as important for education and training *(show and discuss visuals on slide).*

For providers: Keeping these topics in mind (e.g., StUD, StUD medication [side effects, efficacy/effectiveness], addiction as a chronic disease), what are the best ways to educate or train providers on these topics?

a. Probe if needed: What about weekly newsletters, treatment guidelines, YouTube channel, ECHO series?

What are the best ways to educate patients, families, or communities about these topics? *Make sure each of the three groups is discussed if there is time (you may need to probe for this: e.g., “I have heard us talk a lot about families, what about for communities?”)*

a. Probe if needed: What about flyers, commercials, short videos, infographics, websites, information in the mail, social media?

For patients: Keeping these topics in mind (e.g., StUD, StUD medication [side effects, efficacy/effectiveness], addiction as a chronic disease), what are the best ways to educate or share information with patients on these topics?

a. Probe if needed: What about resources in waiting rooms, flyers, commercials, short videos, websites, information in the mail, social media?

What are the best ways to educate providers, families, or communities about these topics? *Make sure each of the three groups is discussed if there is time (you may need to probe for this: e.g., “I have heard us talk a lot about families, what about for communities?”*

a. Probe on examples above if needed.

2.2 What about a website? Let’s take a look at an example (<https://www.vactitoolkit.com/>). *Navigate to the VA website and share your screen. Take a pause on the* *main page and its content. After a beat, scroll down so they can see the testimonials. (“This is a website for care managers who are working with veterans experiencing homelessness. The website has recorded trainings, a roadmap on how to implement, and testimonials.”)*

For providers: Would a website like this be useful for sharing knowledge and resources related to medication treatment for StUD with providers?

a. What about patients, their families, or the community?

For patients: Would a website like this be useful for sharing knowledge and resources related to medication for StUD with patients?

a. What about a website for providers that has similar options (education, testimonials, other resources)?

b. What about a website for families or the community?

**Patient-Centered Care**

3.1 A frequently discussed recommendation was the idea of clinics and care providers prioritizing patient-centered care *(show and discuss visuals on slide).*

For providers: What are some ways you try to build patient-centered care into your practice?

For patients: What are some ways you have experienced patient-centered or individualized care (*Pause here and then explicitly define if needed:* “having treatment options that fit a patient’s individual needs”)?

3.2. Let’s take a look at an example of a tool that can be used to individualize care. (*Introduce the tool and share your screen: This is a template for a personal health inventory that was developed in the veteran system to assist patients in thinking about building a health plan and prioritizing what is important to them*.) <https://www.va.gov/WHOLEHEALTH/docs/PHI_Jan2022_Final_508.pdf>

For providers: (*Pause here to allow them to review*) Can you imagine a tool like this being useful in treating individuals with StUD? Why or why not?

a. What would you change to make it more compatible with your practice?

For patients: (*Pause here to allow them to review*) Can you imagine a tool like this to be useful in treatment for StUD? Why or why not?

a. What would you change to make it more compatible for individuals seeking treatment for StUD?

**Cost and Funding**

4.1 Cost of and funding for medication treatment for StUD was frequently mentioned in interviews as well *(show and discuss visuals on slide).*

For providers: What can clinics and care providers do to assist patients in navigating the cost of treatment for StUD?

a. How can we (researchers) support clinics and care providers in communicating with patients about insurance coverage and medication treatment options?

For patients: In your experience, what resources or people have been helpful in navigating the cost of treatment? (Probe if needed: help with insurance coverage/approvals)

a. What can clinics and care providers do to assist patients in navigating the cost of treatment for StUD?

4.2 For providers only: How often in your clinics do you talk about accessing funding for StUD treatment? What do those conversations look like? (Probe if needed: Through grants, cost-sharing, or raising private funds for StUD treatment?)

a. Who should be at the table when having discussions about new funding sources? (Probe if needed: e.g., what about leadership, external partners, frontline staff?)

**Closing**

5.1 That concludes our discussion for today. Is there anything else you would like to add?
